# Velocity time integral for right upper pulmonary vein in VLBW infants with patent ductus arteriosus

**DOI:** 10.6061/clinics/2016(10)05

**Published:** 2016-10

**Authors:** Gianluca Lista, Silvia Bianchi, Savina Mannarino, Federico Schena, Francesca Castoldi, Mauro Stronati, Fabio Mosca

**Affiliations:** I Ospedale dei Bambini “V. Buzzi”, ICP, Neonatal Intensive Care Unit, Milan, Italy; II IRCCS “Policlinico San Matteo”, Department of Cardiology, Pavia, Italy; III University of Milan, IRCCS “Ca' Granda-Ospedale Maggiore Policlinico”, Neonatal Intensive Care Unit, Milan, Italy; IV IRCCS “Policlinico San Matteo”, Neonatal Intensive Care Unit, Pavia, Italy

**Keywords:** Patent Ductus Arteriosus, Premature Infant, Echocardiography

## Abstract

**OBJECTIVE:**

Early diagnosis of significant patent ductus arteriosus reduces the risk of clinical worsening in very low birth weight infants. Echocardiographic patent ductus arteriosus shunt flow pattern can be used to predict significant patent ductus arteriosus. Pulmonary venous flow, expressed as vein velocity time integral, is correlated to ductus arteriosus closure. The aim of this study is to investigate the relationship between significant reductions in vein velocity time integral and non-significant patent ductus arteriosus in the first week of life.

**METHODS:**

A multicenter, prospective, observational study was conducted to evaluate very low birth weight infants (<1500 g) on respiratory support. Echocardiography was used to evaluate vein velocity time integral on days 1 and 4 of life. The relationship between vein velocity time integral and other parameters was studied.

**RESULTS:**

In total, 98 very low birth weight infants on respiratory support were studied. On day 1 of life, vein velocity time integral was similar in patients with open or closed ductus. The mean vein velocity time integral significantly reduced in the first four days of life. On the fourth day of life, there was less of a reduction in patients with patent ductus compared to those with closed patent ductus arteriosus and the difference was significant.

**CONCLUSIONS:**

A significant reduction in vein velocity time integral in the first days of life is associated with ductus closure. This parameter correlates well with other echocardiographic parameters and may aid in the diagnosis and management of patent ductus arteriosus.

## INTRODUCTION

In the first days of life, pulmonary venous flow significantly changes as the fetal stage proceeds to the neonatal stage. With the onset of spontaneous breathing, the absolute amount of pulmonary blood flow significantly increases, pulmonary vascular resistance decreases and the ductus arteriosus closes 1. In the newborn, pulmonary venous flow velocity dramatically increases at birth due to an increase in total pulmonary flow--it then slightly decreases over the next few days. This reduction in velocity is caused by two factors: 1) a change in the reservoir function of the pulmonary veins and 2) the progressive closure of the ductus in the first hours of life 1,2. In full-term healthy babies, the absolute increase in pulmonary blood flow has a significant effect on the Doppler pattern produced by the pulmonary veins 1.

Pulmonary vein velocity may reflect a sudden increase in pulmonary circulatory volume with additional left-to-right shunting through the ductus arteriosus 2. The relationship between pulmonary vein velocity and pulmonary flow has also been confirmed in previous studies of patients with intraventricular or interatrial defects 3-5.

The changes that occur in pulmonary blood flow in preterm infants at birth have not yet been studied. Very low birth weight (VLBW) infants often present delayed closure of the ductus arteriosus (defined as patent ductus arteriosus, or PDA) with increased pulmonary flow, left cardiac overload with possible heart failure.

For accurate measurement of pulmonary venous flow, blood volume should be measured using velocity time integral (VTI) and diameter used to assess blood volume passing through a vessel; however, such echocardiographic examination is difficult to implement in practice. Preterm infants also frequently exhibit left-to-right shunting through the foramen ovale and therefore relative mitral flow cannot be routinely used as an index of pulmonary venous flow 6.

The VTI of the right upper pulmonary vein (pvVTI) can be used as an index of total pulmonary flow. This parameter is not difficult to measure using two-dimensional images and color Doppler 1. VTI is an independent measure that is not affected by changes in vessel diameter, unlike systolic peak (S) and diastolic peak (D) velocities. However, unfortunately, VTI is also not related to heart rate and it can also be associated with possible inhomogeneous perfusion of the lungs in a newborn. In VLBW infants, early diagnosis and treatment of significant PDA may reduce the risk of clinical deterioration, especially for those requiring mechanical ventilation for respiratory distress.

For this reason, echocardiographic monitoring of ductus flow pattern and cardiac performance is crucial for the detection of significant PDA before deterioration of cardiorespiratory status occurs 7.

The aim of this study was to investigate whether a significant reduction in pvVTI is related to the occurrence of non-significant PDA in VLBW infants on respiratory support during the first 4 days of life. Additionally, the relationship between pvVTI and other echocardiographic parameters routinely used to detect significant PDA was analyzed.

## MATERIALS AND METHODS

### Patients

The current study used a prospective, observational design (local ethical committee approval was obtained) that included three tertiary NICUs in Lombardia-Italy: IRCCS “Policlinico San Matteo” in Pavia, IRCCS “Ca' Granda-Ospedale Maggiore Policlinico”, University of Milan and “V. Buzzi” Ospedale dei Bambini, ICP in Milano.

Eligibility criteria included all inborn infants under 32 weeks of gestational age (24.0-31.6 wks) with a birth weight <1500 gr who were on respiratory support for respiratory failure, intubated and undergoing mechanical ventilation (Assisted Control-AC with Volume Guarantee (VG) option; Vt=5 ml/kg, PEEP level 5 cmH2O) or who were being assisted with NCPAP (NCPAP level 5 cmH2O).

Patients with an antenatal diagnosis of congenital heart disease or surgical lung or abdominal malformations were excluded. Informed consent was obtained from the parents in all cases.

Echocardiographic data were obtained using an Acuson Sequoia® or an Acuson Aspen® scanner with an 8.5 Hz probe, incorporating colour flow, pulsed and continuous wave Doppler ultrasonography. Data were collected on day 1 and day 4 (we considered day one to be the first 24 hours of life and day 4 as >72 and <96 hours of life) by one neonatologist with expertise in echocardiography per center. Acceptable inter-operator variability was obtained (<15% for each parameter based on a pilot population of 10 neonates). Measurements were reviewed by a senior pediatric cardiologist who was blinded to identifying information. The presence of significant PDA at first assessment was treated with prostaglandin inhibitors (indomethacin or ibuprofen). This decision did not interfere with the aim of the study.

Respiratory support was provided following NICU protocols. Respiratory support may interfere with pulmonary flow, mainly due to the airway pressure used during ventilation; therefore, we maintained a constant NCPAP level of 5 cmH2O while using non-invasive support (NCPAP) and a constant PEEP level of 5 cmH2O while using mechanical ventilation.

### Echocardiographic parameters

Pulmonary vein velocity time integral (pvVTI)

Right upper pulmonary vein VTI was measured using pulsed Doppler performed with a four-chamber view. If PDA was present, we evaluated the following:

-Ductus size (DS)

Ductus diameter was assessed from the high left parasternal short-axis view. We standardized this diameter for newborn bodyweight. The PDA diameter was considered significantly increased if >= 1.4 mm/kg 8.

-Flow pattern

Four patterns of PDA shunt flow can be identified using pulsed Doppler echocardiography: pulsatile, growing, closing and pulmonary hypertension patterns 9.

A closing pattern indicates a non-significant PDA, whereas pulsatile and growing patterns indicate a risk of developing clinically significant PDA 10.

Patients with a bi-directional shunt PDA were excluded; this shunt is generally seen in early postnatal life in the presence of high pulmonary vascular resistance. At this developmental stage, PDA shunts are not significant due to the low pressure gradient between the aortic and pulmonary artery.

Patients with a pulmonary hypertension pattern or an undefined flow pattern were excluded from the study. Based on the flow patterns observed on days 1 and 4, we divided the patients into two groups: those with a pulsatile and growing pattern and those with a closing or closed pattern. 

- Left atrium/aortic root diameter ratio (LA/Ao)

M-mode measurement was assessed from the left parasternal long-axis view.

To detect a significant reduction in pvVTI (20% reduction, power 80%), we had to enroll at least 90 infants. Statistical analysis was performed using NCSS for Windows. Normally distributed parametric data were compared using an unpaired Student's t test. The Mann-Whitney U-test was used for non-normally distributed or non-parametric data. Statistical significance was set at *p*<0.05.

## RESULTS

**Figure 1:**
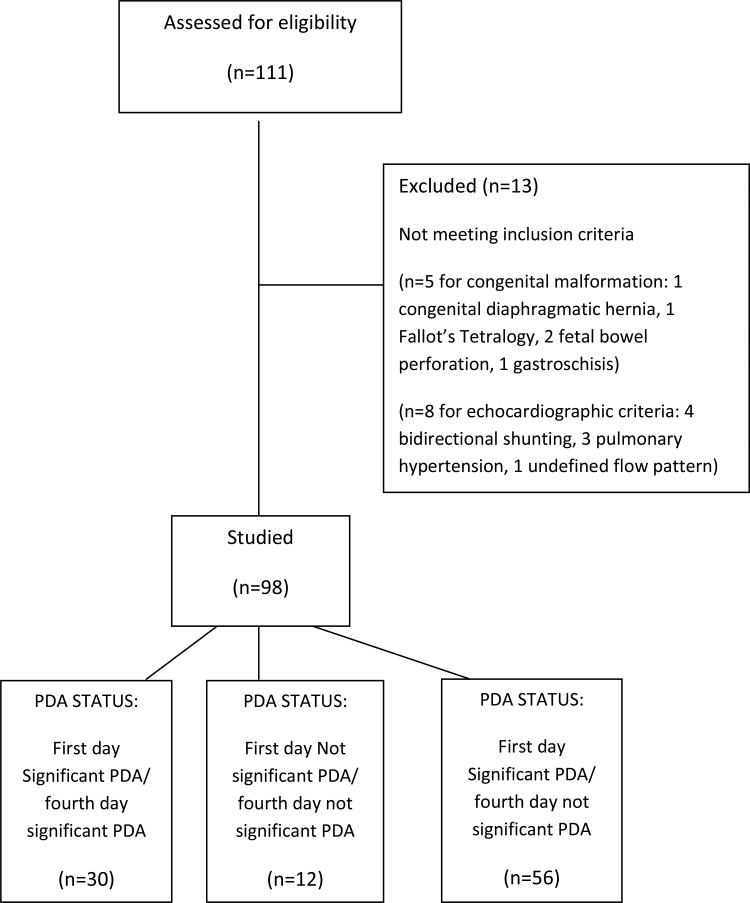
CONSORT diagram showing the flow of participants through echocardiographic PDA evaluation.

A total of 111 inborn premature VLBW infants were consecutively born in our center; see the consort flow diagram in Figure 1 for more information. Of these, 98 patients completed the study with both echocardiographic evaluations.

Clinical characteristics from the first day of life are shown in Table 1.

In total, 46 of the 98 infants were ELBW (BW<1000 g).

The respiratory statuses of the infants on day 4 are described in Table 2.

**Figure 2:**
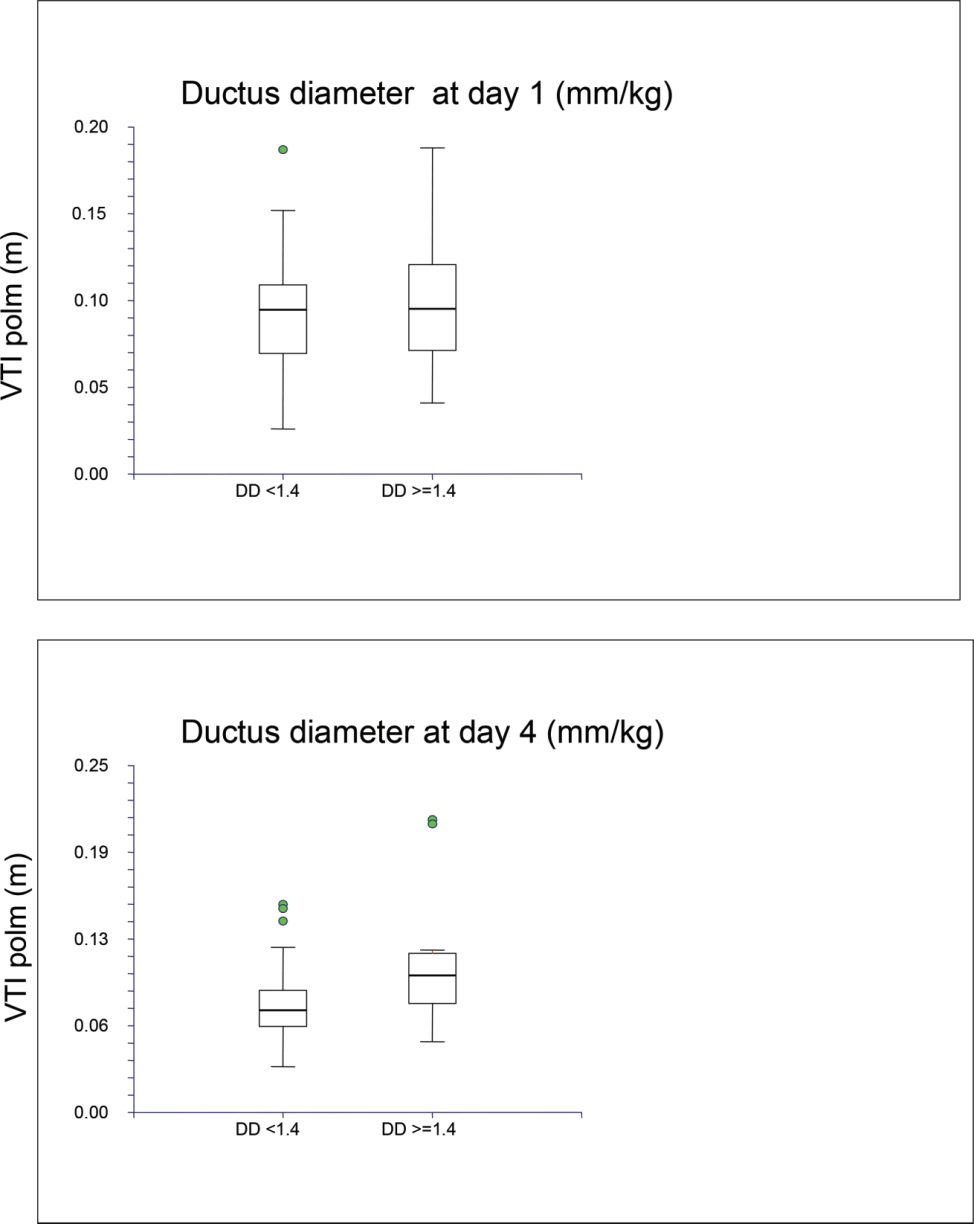
Relationship between pvVTI and ductus diameter on days 1 and 4 of life.

In the whole population, the mean pvVTI showed a statistically significant decline from the first to the fourth day of life (Figure 2). On the first day of life (<24 hours of life), we did not find significant differences in pvVTI between patients with open or closed ductus.

Patients with a significant PDA flow pattern on the first and fourth day of life (significant PDA/significant PDA group, n°30 infants) showed nearly the same pvVTI values, which were high.

Patients with a non-significant PDA flow pattern on the first and fourth day of life (non-significant PDA/non-significant PDA group, n°12 infants) showed a slight decline in pvVTI (Table 3), and their pvVTI values were lower overall.

Patients with a significant PDA flow pattern on the first day of life and a non-significant PDA flow pattern on the fourth day of life (significant PDA/non-significant PDA group, n°56 infants) showed a rapid decline in pvVTI.

### Analysis of correlations between pvVTI and other echocardiographic parameters

- Ductus size

Using a cut-off of 1.4 mm/Kg for ductus diameter, pvVTI was higher in patients with a wide PDA (>1.4 mm/kg BW) compared to patients with a small PDA (<1.4 mm/kg BW) on day 4 but not on day 1 (Figure 2).

- Left atrium/aortic root diameter ratio (LA/Ao)

**Figure 3:**
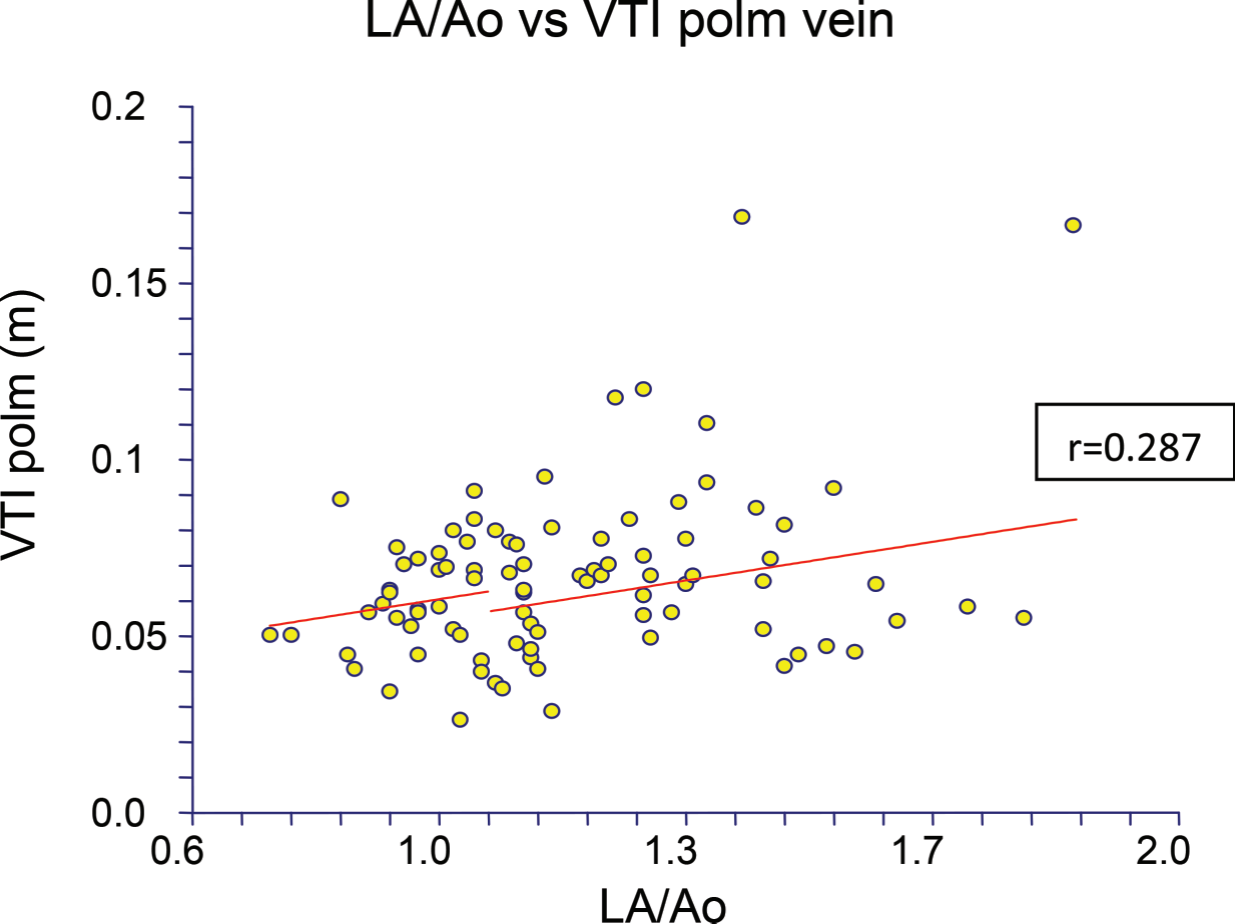
Relationship between pvVTI and LA/Ao on day 4 of life.

Left atrium dimension (expressed with LA/Ao ratio) was correlated with pvVTI only on day 4. For the fourth day of life, there was a weak but statistically significant linear correlation (r=0.287) between LA/Ao ratio and pvVTI (Figure 3).

## DISCUSSION

Large PDAs cause severe worsening of the clinical status of a newborn, particularly by increasing the risk of respiratory distress or pulmonary hemorrhage 11-13.

Identifying and preventing the development of a hemodynamically significant left-to-right shunt through a PDA with increased pulmonary flow is crucial for the management of an affected neonate 14.

Echocardiography has become essential for evaluating significant ductal shunting 15-17. Doppler echocardiographic assessment of PDA shunt flow pattern 10 is useful for predicting the development of significant PDA: a growing and pulsatile Doppler flow pattern indicates the risk of developing a clinically significant PDA, whereas a closing or closed pattern is generally associated with a non-significant shunt.

A pvVTI showing increased pulmonary venous return in the left atrium may reflect a sudden increase in pulmonary circulatory volume with additional left-to-right shunting through the ductus arteriosus 2.

In full-term healthy neonates, Doppler velocities for the pulmonary vein change dramatically during the progression from fetal to neonatal life, probably because of an absolute increase in pulmonary blood flow 1. One hour after birth, without a change in flow pattern, all pulmonary flow velocities dramatically increase. These high velocities show a significant decrease during the first 24 h after birth and a slight decrease three days after birth. pvVTI shows a similar trend.

Changes in pvVTI in preterm infants have not been previously described in the literature.

In our population of preterm infants, pvVTI values decreased during the first days of postnatal life.

On the first day of life, no significant differences were found in pvVTI between infants with an open ductus *vs*. a closed ductus. Considering ductus flow patterns, patients with a pulsatile and growing flow pattern have a significantly higher pvVTI than patients with a closed and closing ductus.

On the fourth day of life, the above difference becomes more evident: patients with a closed and closing ductus have a lower mean pvVTI than patients with a pulsatile and growing flow pattern but have nearly the same pvVTI measurements on days 1 and 4.

We suggest that shunting through the ductus arteriosus modifies pvVTI.

This modification is more evident in cases of significant shunting, particularly on the fourth day of life, as confirmed by other echocardiographic parameters such as ductus size and left atrium-aorta ratio. On day 4, pvVTI correlates with ductus diameter and LA/Ao ratio. Dilatation of the left atrium seems to be an indirect sign of cardiac overload.

One limitation of our study is that we did not focus on correlations between pulmonary flow and gestational age or respiratory status, even though we reported some of these parameters in the results. These investigations were not the aim of our study, although they may be further examined in future studies. Another limitation is that we followed NICU protocols for all infants without varying them between assisted premature infants.

In conclusion, pvVTI seems to be well correlated with other echocardiographic parameters and can help diagnose significant PDA. Further research on the use of pvVTI in a larger group of preterm infants is needed.

## AUTHOR CONTRIBUTIONS

Lista G and Mosca F conceived the study, participated in its coordination and helped draft and review the manuscript. Bianchi S, Mannarino S, Schena F, Castoldi F and Stronati M performed the search of published works, performed the research, contributed to data analysis and helped draft and review the manuscript. All of the authors read and approved the final version of the manuscript.

## Figures and Tables

**Table 1:** Clinical characteristics of enrolled infants in day 1.

Ductus arteriosus	Mean pvVTI (m) 1^st^ day	N	GA (wks)#	BW (g)#	Delivery (Cesarean section/vaginal delivery)	Apgar score 5'#
Non- significant PDA	0.090	12	29±2.7	1061±323	9/3	8±1
Significant PDA	0.095	86	28±1.9	888±289	60/26	7±1
All patients	0.095	98	28±2.5	1007±321	69/29	7±1

# Data are expressed as the mean±SD.

**Table 2:** Respiratory statuses of enrolled infants on day 4 of life.

Group	Non- invasive respiratory supportn (%)	Mechanical ventilation n (%)	Spontaneous breathingn (%)
Significant PDA/Significant PDA (n=30)	7 (23)	18 (60)	5 (16.6)
Non-significant PDA/Non-significant PDA (n=12)	4 (33)	5 (41.6)	3 (25)
Significant PDA/Non-significant PDA (n=56)	15 (26.7)	25 (44.6)	16 (28.5)

Fisher’s exact test = ns

**Table 3:** Changes in pvVTI on the first and fourth days of life in the whole population and in different groups based on PDA evolution.

Ductus arteriosus	n	Mean pvVTI (m)1^st^ day	Mean pvVTI (m)4^th^ day	*p*
Non-significant PDA/non-significant PDA	12	0.090	0.074	0.2
Significant PDA/non-significant PDA	56	0.091	0.073	<0.001
Significant PDA/significant PDA	30	0.103	0.099	0.3
All patients	98	0.095	0.081	<0.001
